# Comparison of Emergency Department Processes and Intensive Care Unit Admissions Between the Local Population and Refugees in Türkiye: A Retrospective Observational Study

**DOI:** 10.3390/healthcare14172855

**Published:** 2026-09-04

**Authors:** Necip Gökhan Güner, Fatih Güneysu, Yusuf Yürümez, Sacit Akdeniz, Nuray Aslan

**Affiliations:** 1 Department of Emergency Medicine, Sakarya University School of Medicine, Sakarya 54100, Türkiye; fatihguneysu55@hotmail.com (F.G.); yyurumez@sakarya.edu.tr (Y.Y.); 2 Department of Emergency Medicine, Sakarya Training and Research Hospital, Sakarya 54100, Türkiye; sacit.akdeniz@gmail.com (S.A.); nurayasanaslan@hotmail.com (N.A.)

**Keywords:** emergency service, equity, health services, Turkey, intensive care units, refugees

## Abstract

**Highlights:**

**What are the main findings?**

- No significant differences were observed between refugees and the local population in examination waiting time or time to first consultation among patients requiring critical care.

**What are the implications of the main findings?**

- Refugee status was associated with higher odds of ICU admission at the study hospital; however, this finding should be interpreted cautiously in light of potential unmeasured clinical and contextual factors.

**Abstract:**

**Background:** This study aimed to compare the emergency department (ED) and intensive care unit (ICU) admission processes of the local population and individuals with migrant, refugee, or asylum status as well as to evaluate differences between the groups. **Methods:** This retrospective observational study was conducted by analyzing the data of 10,163 patients who presented to the Emergency Medicine Clinic of Sakarya Training and Research Hospital between 1 January 2022 and 1 January 2024, and were determined to require ICU care. Among these, 10,060 were from the local population and 103 were refugees. Key variables, such as patients’ demographic characteristics, comorbidities, ED processes, hospital admission, and referral statuses, were collected and analyzed. The data were analyzed using Statistical Package for the Social Sciences (SPSS) version 26.0. **Results:** The mean age of refugee patients was 39.1 ± 26.5 years, and they were younger than the local population. No significant difference was observed between the two groups in terms of examination waiting times and time to the first consultation (*p* = 0.721 and *p* = 0.060, respectively). Refugees were more frequently admitted to the ICU at the study hospital than the local population (85.4% vs. 61.0%, *p* < 0.001). Refugee status remained independently associated with ICU admission at the study hospital after adjustment for age, sex, and major comorbidities (adjusted OR: 2.57, 95% CI: 1.47–4.50; *p* = 0.001). **Conclusions:** In this study, no significant differences were observed between refugees and the local population in examination waiting time, time to first consultation, or ED length of stay. Refugees were younger and had a higher rate of ICU admission at the study hospital. These findings may contribute to a better understanding of healthcare utilization patterns among refugees requiring intensive care in Türkiye.

## 1. Introduction

The number of individuals displaced because of conflict, violence, or persecution has reached record levels globally. Türkiye stands out as one of the countries hosting the largest refugee populations worldwide. As of November 2024, Türkiye hosts approximately 3.2 million registered Syrians and an additional 222,000 individuals of other nationalities under the “Temporary Protection” program [1]. When unregistered migrants and asylum seekers are included, this number is estimated to reach 5 million [2].

For refugees, as with the local population, emergency departments (EDs) often serve as the initial point of contact for healthcare, with some cases requiring hospital ward or intensive care unit (ICU) admission [3]. Patients with ICU admission indications generally include those in critical condition requiring urgent intervention, but the limited capacity of ICU beds poses a significant challenge. According to OECD Health Statistics, Türkiye had 39.6 adult intensive care beds per 100,000 population in 2021, one of the highest rates among the OECD countries included in the analysis [4]. In addition, studies highlight that refugees face barriers in accessing healthcare services, including language and cultural differences, as well as economic hardships [3,5,6]. Population mobility and migration may also have broader implications for healthcare systems by contributing to changes in disease epidemiology and patterns of healthcare demand. Recent observations of changing disease patterns in Türkiye further illustrate the potential healthcare challenges associated with evolving demographic and population dynamics [7,8]. For these reasons, ensuring an equitable and fair approach regarding access to ICU services for refugees is of paramount importance [9]. Moreover, the accessibility and equity of healthcare services provided to refugees are considered crucial benchmarks for evaluating the overall performance of the healthcare system [10].

Although several studies have examined healthcare utilization and ED visits among refugees, comparative data regarding ED process metrics among critically ill refugees and local population requiring intensive care unit admission remain limited. This study aims to compare ED process metrics between critically ill refugees and local population requiring intensive care unit admission. In particular, selected emergency care process indicators, such as waiting time for examination, time to first consultation, length of ED stay, and admission or referral outcomes, have been insufficiently investigated in this population. Evaluating these process measures may provide a more objective assessment of whether refugee status is associated with differences in time-sensitive emergency and critical care processes.

The aim of this study is to compare ED process indicators and intensive care unit admission outcomes between refugees and the local population who presented to the ED and required intensive care in Türkiye. Selected ED process indicators relevant to access and care delivery were evaluated, including waiting time for examination, time to initial consultation, length of ED stay, and admission or referral status.

## 2. Materials and Methods

This retrospective observational study was conducted by analyzing the data of patients who presented to the Emergency Medicine Clinic of Sakarya Training and Research Hospital between 1 January 2022 and 1 January 2024, and were determined to require ICU care.

Patients included in the study were identified through the Hospital Information Management System, and the necessary patient information was recorded for analysis. Inclusion and exclusion criteria were clearly defined to ensure the homogeneity of the study group and enhance the reliability of the analyses.

Within the scope of the study, key variables such as demographic characteristics (gender and age), comorbidities (hypertension [HT], chronic kidney disease [CKD], chronic obstructive pulmonary disease [COPD], asthma, diabetes mellitus [DM]), ED processes (examination waiting time, initial consultation time, total number of consultations, and length of ED stay), hospital admission, and referral statuses were analyzed.

The confidentiality of personal data was ensured, and all analyses were performed using anonymized data. Before initiating the study, approval was obtained from the Sakarya University Ethics Committee (IRB No: E-43012747-050.04-438097), and permission was obtained from the hospital administration.

Refugee status was determined from official registration records in the national hospital information system. Individuals registered under temporary protection, refugee, or asylum-seeker status were classified as refugees for the purposes of this study. Also, ICU admission decisions were based on routine clinical assessment by emergency physicians and relevant consulting specialists, in accordance with institutional practice and national critical care admission principles.

The primary outcome was ICU admission at the study hospital. This was defined as admission to an intensive care unit within the study hospital after the indication for ICU care had been established in the ED. Patients for whom an ICU indication was established in the study ED but who were transferred to an intensive care unit at another healthcare facility were classified as referrals. Thus, the primary outcome used in the logistic regression analysis distinguished ICU admission at the study hospital from referral to another institution for ICU care.

Inclusion Criteria:

- Patients requiring ICU admission as determined by an emergency medicine physician or relevant specialist consultant.
- Patients identified as local population or refugees.
- Patients whose initial assessment and ICU indication were finalized in the ED.
- Patients aged ≥18 years.
- Patients with complete medical records.

Exclusion Criteria:

- Patients referred to the ED from another healthcare facility.
- Patients whose ICU admission indication was established in outpatient clinics or non-ED units.
- Patients eligible for ICU admission but not admitted because of treatment refusal.
- Patients with indications for palliative care.
- Nonrefugee foreign nationals or those categorized as tourists.

Patients referred from other healthcare facilities were excluded because ED process indicators, including waiting times, consultation intervals, and length of stay, could be substantially influenced by pre-hospital evaluation and management at the referring institution. Patients with ICU admission decisions established outside the ED were excluded to ensure that all analyzed process metrics reflected care delivered within the study ED. Patients with incomplete records were excluded because the primary study variables could not be reliably assessed. Patients with palliative care indications were excluded because treatment goals and admission decisions in this population are often based on end-of-life care considerations rather than standard intensive care management, which could substantially affect ED processes and ICU admission or referral outcomes.

**Figure 1:**
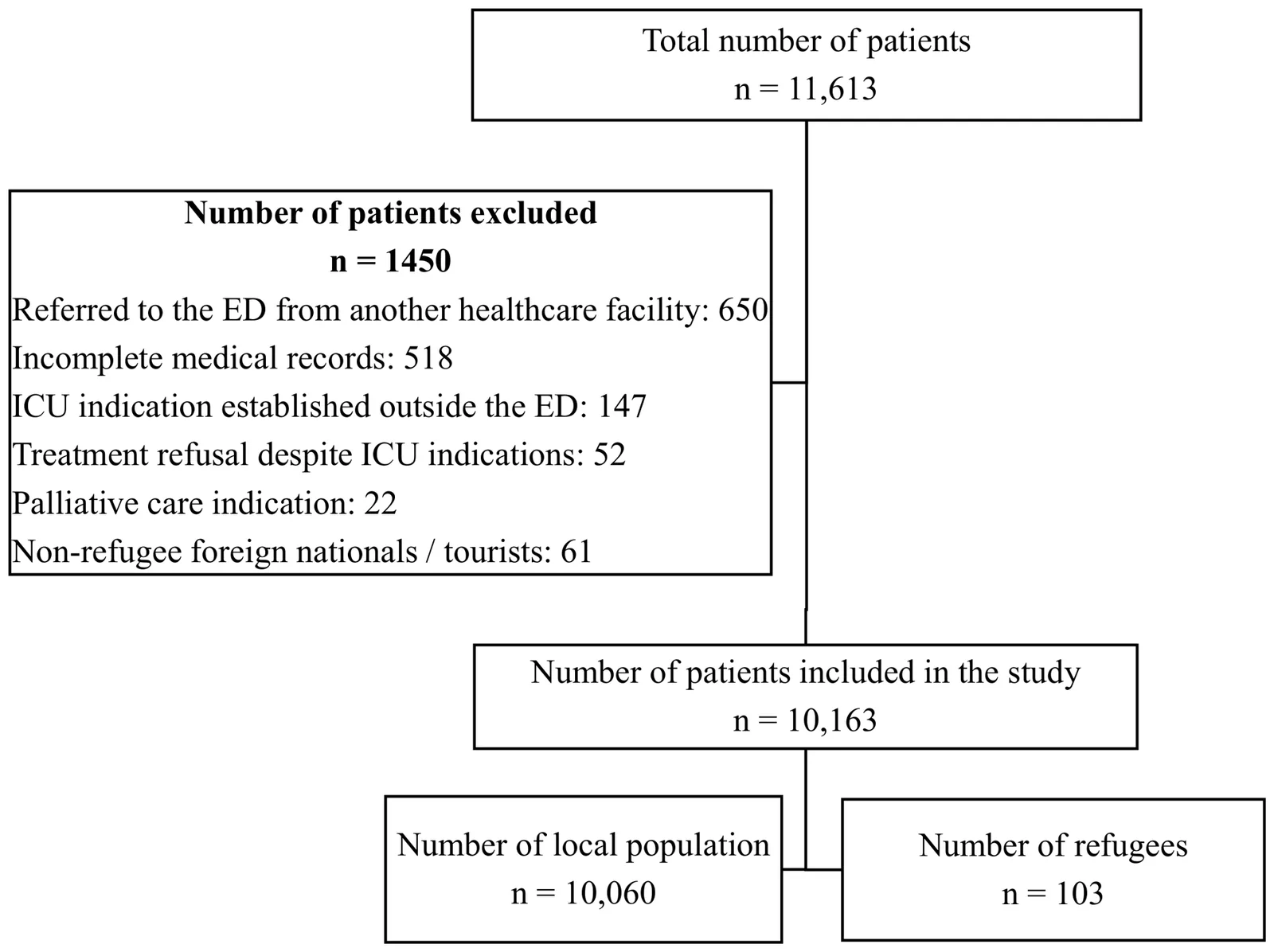
Study flow chart.

A total of 11,613 patients evaluated for ICU admission in the ED were initially identified. Of these, 1450 patients were excluded according to the predefined exclusion criteria, leaving 10,163 patients for the final analysis. Of the included patients, 103 were refugees, and 10,060 were local population (Figure 1).

### Statistical Analysis

The data were analyzed using the Statistical Package for the Social Sciences (SPSS) version 26.0. Categorical variables were presented as frequencies and percentages. Normally distributed continuous variables were expressed as mean ± standard deviation (SD), whereas non-normally distributed continuous variables were presented as median and interquartile range (IQR). Normality was evaluated using the Kolmogorov–Smirnov test and visual assessment of the distributions. Group comparisons were performed using the chi-square test for categorical variables, the independent-samples *t*-test for normally distributed continuous variables, and the Mann–Whitney U test for non-normally distributed continuous variables. For non-normally distributed continuous variables, between-group effect estimates were additionally expressed as Hodges–Lehmann location shift estimates with 95% confidence intervals.

To identify independent predictors of ICU admission at the study hospital, univariable and multivariable logistic regression analyses were conducted. The multivariable model included demographic factors (age, sex) and major comorbidities (hypertension, chronic kidney disease, chronic obstructive pulmonary disease, asthma, and diabetes mellitus), selected for their clinical relevance to critical care outcomes. Prior to multivariable modeling, multicollinearity among independent predictors was assessed using variance inflation factor (VIF) and tolerance values. All VIF values were below 1.13 and all tolerance values were above 0.88, indicating no evidence of problematic multicollinearity. Missing data were managed using complete-case analysis based on predefined exclusion criteria. Among the 10,163 patients included in the final study population, complete data were available for all variables entered into the logistic regression model; therefore, all 10,163 patients were included in the complete-case regression analysis. Model calibration and goodness of fit were evaluated using the Hosmer–Lemeshow test. Regression outcomes are reported as Odds Ratios (ORs) with 95% Confidence Intervals (CIs). All tests were conducted with a two-tailed significance level of 5%, and a *p*-value < 0.05 was considered statistically significant.

## 3. Results

Table 1 summarizes the demographic characteristics, comorbidities, and ED process indicators of local population and refugees requiring ICU admission. Among the patients included in the study, males constituted the majority in both groups (60.2% and 58.6%, respectively; *p* = 0.750). Refugees were significantly younger than the local population (39.1 ± 26.5 vs. 64.5 ± 18.6 years, *p* < 0.001). Hypertension was less frequent among refugees (10.7% vs. 30.7%, *p* < 0.001), whereas no refugee had a documented asthma diagnosis. No significant differences were observed between the groups in examination waiting time (*p* = 0.721; Hodges–Lehmann estimate: 0 min, 95% CI: −1 to 1), time to first consultation (*p* = 0.060; Hodges–Lehmann estimate: 6 min, 95% CI: 0 to 20), or emergency department length of stay (*p* = 0.056; Hodges–Lehmann estimate: 38 min, 95% CI: −1 to 80). The total number of consultations differed significantly between the groups (*p* = 0.008, Hodges–Lehmann estimate: 0, 95% CI: 0 to 1). ICU admission at the study hospital was more frequent among refugees than among the local population in the unadjusted analysis (85.4% vs. 61.0%, *p* < 0.001).

Univariable and multivariable logistic regression analyses were performed to identify factors associated with ICU admission at the study hospital. In the univariable analysis, refugee status was significantly associated with higher odds of ICU admission at the study hospital (OR: 3.76, 95% CI: 2.17–6.50, *p* < 0.001). After adjustment for age, sex, hypertension, chronic kidney disease, chronic obstructive pulmonary disease, asthma, and diabetes mellitus, refugee status remained independently associated with ICU admission at the study hospital (adjusted OR: 2.57, 95% CI: 1.47–4.50, *p* = 0.001). The overall model demonstrated acceptable calibration according to the Hosmer–Lemeshow goodness-of-fit test (χ^2^ = 15.01, *p* = 0.059) (Table 2).

## 4. Discussion

In recent years, Türkiye has experienced a significant influx of refugees, particularly because of the civil war in Syria. This situation has placed a considerable burden on the national healthcare system, particularly on EDs [8,11]. This exceptional situation has raised concerns about equitable access to healthcare and service delivery for refugee patients, while highlighting the risk of insufficient access to healthcare and potential loss of rights for the local population.

Among patients requiring ICU admission, the present study identified several important differences and similarities between refugees and the local population. Refugee patients were significantly younger, while no significant differences were observed between the groups in examination waiting time, time to first consultation and length of ED stay. In contrast, refugees had fewer consultations. Refugees were more frequently admitted to the ICU at the study hospital, and refugee status remained significantly associated with higher odds of ICU admission at the study hospital after adjustment for age, sex, hypertension, chronic kidney disease, chronic obstructive pulmonary disease, asthma, and diabetes mellitus. However, this association should be interpreted cautiously because primary diagnosis, illness severity, referral alternatives, and other potentially relevant clinical and contextual factors could not be fully accounted for.

Previous studies have shown that ICU admissions are more frequent among males. This is attributed to various social, economic, physical, biological, and behavioral factors as well as men’s greater exposure to physical trauma, comorbidities, and lifestyle risks [12,13,14]. In addition, previous studies have reported that even when women have more comorbidities, men are admitted to ICUs more frequently [14,15]. Consistent with these observations, male sex was independently associated with higher odds of ICU admission at the study hospital in the present study. Refugee populations requiring emergency and critical care generally have a relatively young demographic profile. In a multicenter study of refugees requiring ICU admission for community-acquired infections in Türkiye, the mean age was 45.92 ± 20.16 years [16]. Consistent with this observation, the present study also found that refugee patients requiring ICU care were significantly younger than the local population.

Although disease severity is generally associated with comorbidities and overall health status, comorbidities in refugees remain underexplored in the literature [16,17]. Existing studies have primarily focused on mental health issues and infectious diseases. However, one study reported a high prevalence of comorbidities among refugees, with 9.5% having three or more concurrent conditions [18]. In the present study, several documented comorbidities were less frequent among refugee patients. This finding may partly reflect the younger age profile of the refugee group; however, differences in documentation and other unmeasured factors cannot be excluded.

Overcrowding in EDs is a critical and growing issue in public hospitals worldwide [19]. A study conducted in Türkiye demonstrated that the increasing influx of refugees has contributed to ED overcrowding [11]. Overcrowding naturally influences factors such as the length of ED stay, waiting times, consultation request times, and ICU admission rates [19]. Derlet and Richards identified language and cultural barriers among the factors contributing to emergency department overcrowding and prolonged care processes [20]. Although the ED where the present study was conducted was crowded, no significant differences were found between the local population and refugee patients in terms of waiting times for examination or the first consultation request. No difference was found in the length of stay of refugee patients in the ED. No statistically significant differences were observed between refugees and the local population in waiting time for examination or time to first consultation. These findings suggest broadly similar patterns in the selected ED process indicators evaluated in this study; however, they should be interpreted with caution in light of the study design, group size imbalance, and the potential influence of unmeasured clinical and contextual factors. Our findings should therefore be interpreted narrowly as showing similarity in selected measured ED process indicators rather than demonstrating equitable healthcare delivery overall.

Maintaining equity, particularly in access to healthcare, is crucial considering the economic disparities that may arise. Financial barriers, including healthcare costs and out-of-pocket payments, may limit refugees’ access to healthcare services [3,21,22]. In the present study, refugees requiring ICU care were more frequently admitted to the ICU at the study hospital. However, the retrospective design does not allow the underlying reasons for these differences to be determined. Factors such as disease severity, primary diagnosis, ICU bed availability, referral logistics, patient preferences, socioeconomic circumstances, and other unmeasured clinical or social characteristics may have influenced these outcomes.

### Limitations

This study sheds light on healthcare utilization and demographic differences between the local population and refugees in Türkiye. Although the broad dataset and the comparison of healthcare use between two distinct demographic groups are key strengths, the study has several limitations. First, the single-center and retrospective design inherently limited control over data quality, completeness, and variable selection. Because the analyses were based on routinely recorded medical data, several potentially important confounding factors could not be evaluated, including socioeconomic status, educational level, language proficiency, availability and use of interpreter services, health literacy, prehospital characteristics, triage categories, primary diagnosis and other social determinants of health. These factors may influence access to care, communication with healthcare professionals, ED processes, and ICU admission at the study hospital or referral decisions.

Second, there was a substantial imbalance in group sizes, with only 103 refugees compared with 10,060 individuals from the local population. Although no sampling procedure was applied and all eligible ICU admissions during the study period were included, meaning that this distribution reflects the actual composition of ICU admissions in the study population, the relatively small number of refugees remains an important limitation. This imbalance may have reduced the statistical precision of estimates involving the refugee group and limited the ability to detect smaller between-group differences. Consequently, comparisons involving refugees should be interpreted with caution, particularly for outcomes with modest effect sizes. Larger multicenter studies including a greater number of refugee patients are needed to confirm the robustness and generalizability of these findings. Third, excluding patients with incomplete records and those referred from other healthcare facilities may have introduced selection bias. Since the characteristics of excluded patients were not analyzed separately, the potential impact of these exclusions on comparisons between refugee and local populations cannot be fully determined. In addition, standardized severity scores such as APACHE II or SOFA were not routinely recorded and therefore could not be incorporated into the analysis. Consequently, differences in disease severity between refugee and local populations could not be fully assessed or adjusted for in the analyses. In addition, the study population consisted exclusively of patients who were deemed eligible for ICU admission. Therefore, the findings cannot be used to draw conclusions regarding ED utilization or ICU admission eligibility among refugees and the local population. Finally, this was a single-center study conducted at a tertiary referral hospital. Therefore, the findings may not be fully generalizable to other healthcare settings, regions, or countries with different healthcare systems and refugee populations.

## 5. Conclusions

Among patients requiring ICU care, no significant differences were observed between refugees and the local population in examination waiting time, time to first consultation, or ED length of stay. However, refugees had fewer consultations and a higher rate of ICU admission at the study hospital. The association between refugee status and ICU admission at the study hospital remained significant after adjustment for age, sex, and major comorbidities. These findings should be interpreted cautiously because important clinical and social factors, including disease severity, triage category, primary diagnosis, and socioeconomic characteristics, could not be fully accounted for. Further multicenter studies incorporating these variables are needed to better understand differences in emergency and critical care processes between refugee and local populations.

## Figures and Tables

**Table 1:** Demographic and Clinical Information of Patients Included in the Study.

Categories		Refugees (*n* = 103)	Local Population (*n* = 10,060)	*p*-Value
Sex, *n* (%)	Female	41 (39.8)	4161 (41.4)	0.750
	Male	62 (60.2)	5899 (58.6)	
Age, years; mean (SD)		39.1 (26.5)	64.5 (18.6)	<0.001
Comorbidities, *n* (%)	Hypertension	11 (10.7)	3088 (30.7)	<0.001
	CKD	1 (1.0)	262 (2.6)	0.299
	COPD	11 (10.7)	1281 (12.7)	0.534
	Asthma	0 (0.0)	480 (4.8)	0.023
	DM	0 (0.0)	112 (1.1)	0.282
Waiting time for examination, min; median (IQR)		4 (1–8)	4 (2–7)	0.721
Time to first consultation, min; median (IQR)		68 (1–128)	77.5 (19–133)	0.060
Total number of consultations, *n*; median (IQR)		1 (1–3)	2 (1–3)	0.008
Length of ED stay, min; median (IQR)		253 (106–383)	266 (150–440)	0.056
Index of Admission to Study Hospital ICU and Referral to Another ICU, *n* (%)	Study Hospital	88 (85.4)	6133 (61.0)	<0.001
	Referral	15 (14.6)	3927 (39.0)	

Abbreviations: CKD, Chronic kidney disease; COPD, Chronic obstructive pulmonary disease; DM, Diabetes mellitus; ICU, Intensive care unit; SD, Standard deviation.

**Table 2:** Univariable and Multivariable Logistic Regression Analyses of Factors Associated with ICU Admission at the Study Hospital.

Variables	Univariable OR	95% CI	*p*	Adjusted OR	95% CI	*p*
Refugee status	3.76	2.17–6.50	<0.001	2.57	1.47–4.50	0.001
Age	0.98	0.978–0.983	<0.001	0.98	0.97–0.99	<0.001
Sex (Male) *	1.61	1.48–1.74	<0.001	1.44	1.33–1.57	<0.001
HT	0.91	0.84–1.00	0.042	1.12	1.03–1.23	0.013
CKD	1.18	0.92–1.53	0.200	1.28	0.98–1.67	0.065
COPD	0.96	0.86–1.09	0.547	1.05	0.93–1.19	0.444
Asthma	0.78	0.65–0.94	0.008	0.91	0.74–1.10	0.326
DM	0.65	0.45–0.95	0.025	0.69	0.47–1.01	0.058

Abbreviations: CKD, Chronic kidney disease; COPD, Chronic obstructive pulmonary disease; DM, Diabetes mellitus; OR: Odds Ratio; CI: Confidence Interval. * For sex, female was used as the reference category.

## Data Availability

The datasets used and/or analyzed during the current study are available from the corresponding author on reasonable request.

## Abbreviations

ED	Emergency department
ICU	Intensive care unit
SD	Standard deviation
SPSS	Statistical Package for the Social Sciences
